# Draft Genome Assemblies of Five Robust Yarrowia lipolytica Strains Exhibiting High Lipid Production, Pentose Sugar Utilization, and Sugar Alcohol Secretion from Undetoxified Lignocellulosic Biomass Hydrolysates

**DOI:** 10.1128/MRA.01040-18

**Published:** 2018-09-27

**Authors:** Caleb Walker, Seunghyun Ryu, Hyunsoo Na, Matthew Zane, Kurt LaButti, Anna Lipzen, Sajeet Haridas, Kerrie Barry, Igor V. Grigoriev, Joshua Quarterman, Patricia Slininger, Bruce Dien, Cong T. Trinh

**Affiliations:** a Department of Chemical and Biomolecular Engineering, The University of Tennessee, Knoxville, Tennessee, USA; b U.S. Department of Energy, The Joint Genome Institute, Walnut Creek, California, USA; c The National Center for Agricultural Utilization Research, Peoria, Illinois, USA; University of California, Riverside

## Abstract

Screening the genetic diversity of 45 Yarrowia lipolytica strains identified five candidates with unique metabolic capability and robustness in undetoxified switchgrass hydrolysates, including superior lipid production and efficient pentose sugar utilization. Here, we report the genome sequences of these strains to study their robustness and potential to produce fuels and chemicals.

## ANNOUNCEMENT

Yarrowia lipolytica is a dimorphic, generally regarded as safe (GRAS) oleaginous budding yeast (subphylum *Saccharomycotina*). It possesses unique phenotypes, including hydrocarbon assimilation (1–5), specialty lipid and organic acid production (6–11), and resistance to harsh environments, including high salinity (12), broad-range pH (13), and ionic liquid (14). By screening a comprehensive set of 45 Y. lipolytica strains with genetic diversity from the Agricultural Research Service Culture Collection (https://nrrl.ncaur.usda.gov/), we identified five promising candidate strains, YB-392, YB-419, YB-420, YB-566, and YB-567, exhibiting beneficial phenotypes for industrial biocatalysis, including biomass hydrolysate consumption, inhibitor tolerance, and lipid and fatty acid production (15). In this study, we sequenced the genomes of these robust Y. lipolytica strains to aid further research into their physiology, metabolism, and genetics as well as metabolic engineering and synthetic biology for industrial biocatalysis.

The genomes of five Y. lipolytica isolates were extracted with the Zymo Research fungal/bacterial DNA miniprep kit (catalog number D6005; Zymo Research, Irvine, CA). Sequencing was carried out by The Department of Energy Joint Genome Institute (DOE JGI) using Illumina 500-bp insert size fragments, for which 100 ng of DNA was sheared to 500 bp using the LE220 focused ultrasonicator (Covaris, Woburn, MA) and size selected using solid phase reversible immobilization (SPRI) beads (Beckman Coulter, Brea, CA). The fragments were treated with end repair, A tailing, and ligation of Illumina-compatible adapters (IDT, Inc., Skokie, IL) using the KAPA-Illumina library creation kit (Kapa Biosystems, Boston, MA). All prepared libraries were quantified using the Kapa Biosystems next-generation sequencing library quantitative PCR (qPCR) kit and run on a Roche LightCycler 480 real-time PCR instrument. The quantified libraries were then multiplexed with other libraries, and the pool of libraries was then prepared for sequencing on the Illumina HiSeq sequencing platform utilizing a TruSeq paired-end cluster kit v4 and Illumina’s cBot instrument to generate a clustered flow cell for sequencing. Sequencing of the flow cell was performed on the Illumina HiSeq 2500 sequencer using the HiSeq TruSeq sequencing by synthesis (SBS) kits v4 following a 2 × 100-bp indexed run recipe. All raw Illumina sequence data were filtered for artifact/process contamination using the JGI quality control (QC) pipeline. Briefly, BBDuk v36.94 (http://bbtools.jgi.doe.gov) was used to remove contaminants, reads that contained adapter sequences, and right quality trim reads where quality dropped to 0. BBDuk was also applied to eliminate reads containing 1 or more “N” bases, having an average quality score across the read of less than 13 or containing a minimum length of ≤ 41 bp or 33% of the full read length. Using BBMap, reads that were mapped to masked human, cat, dog, and mouse references at 95% identity and aligned to common microbial contaminants were separated. Filtered genomic reads were assembled with SPAdes v3.11.1 (16) using the parameters –phred-offset 33 –cov-cutoff auto -t 16 -m 115 –careful –12 to produce the target nuclear assembly. All genomes were annotated with the reference genome FKP355 (https://genome.jgi.doe.gov/Yarlip1) using the JGI annotation pipeline (17), which integrates an array of tools for gene prediction, annotation, and analysis (18).

### Data availability.

The whole-genome assemblies and annotation were deposited at DDBJ/EMBL/GenBank under the accession numbers listed in Table 1. The versions provided in this paper are the first versions.

**TABLE 1:** Whole-genome assemblies and annotation for five *Yarrowia lipolytica* strains

Strain	GenBank accession no.	SRA no.	BioProject no.	Genome size (Mbp)	Coverage (×)	No. of contigs	No. of gene models
YB392	QPFG00000000	SRP129817	PRJNA370154	20.18	735	362	6,750
YB419	QPFH00000000	SRP129816	PRJNA370155	20.14	740	370	6,751
YB420	QPFI00000000	SRP129815	PRJNA370156	20.2	259	369	6,772
YB566	QPFJ00000000	SRP129822	PRJNA370157	20.27	148	271	6,764
YB567	QPFK00000000	SRP129827	PRJNA370158	20.27	756	263	6,776
